# A Rapid In-Clinic Test Detects Acute Leptospirosis in Dogs with High Sensitivity and Specificity

**DOI:** 10.1155/2016/3760191

**Published:** 2016-03-24

**Authors:** Angeli Kodjo, Christophe Calleja, Michael Loenser, Dan Lin, Joshua Lizer

**Affiliations:** ^1^Laboratoire des Leptospires, VetAgro Sup, Campus Veterinaire de Lyon, 1 avenue Bourgelat, 69280 Marcy l'Etoile, France; ^2^Zoetis Global Diagnostics, 100 Campus Drive, Florham Park, NJ 07932, USA; ^3^Zoetis Veterinary Medicine Research & Development, Hoge Wei 10, 1930 Zaventem, Belgium; ^4^Zoetis Veterinary Medicine Research & Development, 333 Portage Street, Kalamazoo, MI 49007, USA

## Abstract

A rapid IgM-detection immunochromatographic test (WITNESS® Lepto, Zoetis) has recently become available to identify acute canine leptospirosis at the point of care. Diagnostic sensitivity and specificity of the test were evaluated by comparison with the microscopic agglutination assay (MAT), using a positive cut-off titer of ≥800. Banked serum samples from dogs exhibiting clinical signs and suspected leptospirosis were selected to form three groups based on MAT titer: (1) positive (*n* = 50); (2) borderline (*n* = 35); and (3) negative (*n* = 50). Using an analysis to weight group sizes to reflect French prevalence, the sensitivity and specificity were 98% and 93.5% (88.2% unweighted), respectively. This test rapidly identifies cases of acute canine leptospirosis with high levels of sensitivity and specificity with no interference from previous vaccination.

## 1. Introduction

Leptospirosis is a global bacterial zoonotic disease affecting humans and wild and domestic animals, including dogs. Clinical signs of leptospirosis in dogs are nonspecific, typically including renal and/or hepatic dysfunction, and can manifest as subclinical, chronic, or acute infections, sometimes with fatal outcomes. Infected animals excrete* Leptospira* in their urine and can pose a risk to humans who become infected through damaged skin or via the conjunctiva or mucosa, causing a potentially fatal disease [1].

The reference serological test for diagnosis is the microscopic agglutination test (MAT). Because the test is complex to perform and interpret, serum must be sent to a reference laboratory [1]. As such, MAT tends to be the last resort in first-opinion practice. An immunochromatographic test (WITNESS Lepto, Zoetis) has been developed, offering the advantage of a result in 10 min. The test uses whole cell antigen extracts of* L. kirschneri* serovar Grippotyphosa and* L. interrogans* serovar Bratislava to detect canine IgM made in response to infection. These serovars were selected as they are of clinical relevance and relative prevalence worldwide [2–8]. However, unpublished data from internal studies and the current study suggest broad serovar reactivity on the test, likely due to conserved antigens across pathogenic* Leptospira*. The objective of the present study was to evaluate the sensitivity (Se) and specificity (Sp) of this new test in client-owned French dogs as compared to MAT.

## 2. Materials and Methods

All samples in this study were taken from an established bank of clinical samples submitted by French veterinarians to the Laboratoire des Leptospires (VetAgro Sup, Marcy L'Etoile, France) for diagnostic testing because leptospirosis was suspected. A total of 135 canine serum samples were tested using WITNESS Lepto retrospectively compared to MAT. Based on previous MAT results, the samples were divided into three groups: (1) a positive group (*n* = 50) defined as having a MAT titer ≥800 for any of the 19 serovars used in the test and a diagnosis of leptospirosis; (2) a borderline group (*n* = 35) defined as having a MAT titer <800 for at least one nonvaccine serovar and an inconclusive diagnosis; and (3) a negative group (*n* = 50) defined as having MAT titers completely negative or titers ≤400 only for vaccine serovars and a negative diagnosis of leptospirosis. The samples were tested in a random order and the technician performing the test was blinded. Each sample was tested using a prototype of WITNESS Lepto, following the manufacturer's instructions. To perform the test, 5 *μ*L of sample was added to the sample well of the test cassette. Four drops (~140 *μ*L) of a chase buffer was then added and the test was allowed to develop for 10 minutes, at which time the result was interpreted. The formation of only a red control line indicates a negative test, while the formation of a red test line and control line indicates a positive test.

Because the samples in each group were selected to ensure a sufficient sample size, the distribution of the samples (positive, borderline, and negative) in this study did not reflect the empirically determined prevalence in France [7]. To adjust for this in the calculation of Se and Sp, the group sample sizes were weighted to reflect the prevalence rate reported by Renaud et al., 2013 (Table 1). The weighting was achieved by multiplying the French prevalence for each group by the total *n* (*n* = 135). The percentage of true and false positives or negatives per group was calculated using the original study data. The percentages were then applied to the weighted groups for the purposes of Se and Sp calculations.

Diagnostic Se and Sp of WITNESS Lepto were evaluated by comparison with MAT, using a positive cut-off titer of ≥800. All the samples in the positive group were defined as positive, whereas the samples in the borderline and negative groups were defined as negative. The analysis determined Se and Sp with 95% Jeffreys' confidence intervals (CI) [9, 10].

## 3. Results and Discussion

Forty-nine of 50 (49/50) samples in the MAT positive group were positive, while 25/35 MAT borderline samples and all 50 MAT negative samples were negative on WITNESS Lepto (Table 2(a)). Using the weighted distribution, Se was 98% (95% CI 88.7 to 99.9%) and Sp was 93.5% (95% CI 87.4 to 97.1%). With no weighting to the sample distribution, the Se remained at 98% and the Sp was 88.2%. The data is summarized in Table 2(b). Forty-two of the 50 MAT negative dogs had previously received a* Leptospira* vaccine with 38 of them being within a year of vaccination, suggesting that previous vaccination did not interfere with a correct test result.

The level of agreement between WITNESS Lepto and MAT was very high for samples that were either MAT positive or MAT negative. All but one of the samples where the WITNESS result differed with MAT came from the borderline group. The borderline samples were considered negative as their MAT titers were not ≥800. With this classification, some samples in the borderline group were still likely to be truly infected. An IgM immunoblot assay had 88% sensitivity in the first three days of human leptospirosis, compared to 2% sensitivity for the MAT [11]. Another human IgM ELISA detected infection in 29% of the cases before MAT had detectable titers [12]. Thus, WITNESS Lepto may have been correctly identifying positive samples in the borderline group before they reached a MAT titer of ≥800. This emphasizes the difficulty in the interpretation of a single MAT in practice. The sensitivity and specificity of MAT increase considerably when samples collected from the animal one or two weeks apart can be tested [13]. However, client compliance can be difficult due to an extra trip to the clinic at extra cost. The WITNESS test provides a time-saving, easy to interpret, and economical solution in this regard. The test provides additional advantages to the MAT because the test is safe and does not require working with live organism, as is the case with the MAT. The convenience of testing in the clinic provides immediate identification of infected dogs, allowing for proper quarantine and handling protocols to be implemented which reduces the risk of transmission to humans and other animals. Whereas the MAT detects both IgM and IgG, WITNESS Lepto detects only IgM, simplifying result interpretation and negating cross-reactivity with IgG antibody from dogs with a previous* Leptospira* vaccination [14].

## 4. Conclusions

In conclusion, this study indicates that WITNESS Lepto is a reliable test with high levels of sensitivity and specificity, as compared to the MAT, for the diagnosis of acute leptospirosis in dogs that are showing compatible clinical signs. With its ease of use and immediate result, it can transform the veterinarian's case management by enabling earlier diagnosis and treatment or ruling out of leptospirosis, all to the benefit of both the patient and the public health.

## Figures and Tables

**Table 1:** Weighted prevalence rates based on French data^a^.

Group	*n* in present study	French prevalence^a^	Weighted *n* in present study
Positive (MAT titer ≥ 800)	50	25%	33.75
Borderline (MAT titer < 800)	35	17%	22.95
Negative (MAT titer negative or vaccinal ≤ 400)	50	58%	78.3

MAT: microscopic agglutination test.

^a^Renaud et al., 2013 [7].

**Table:** (a) Unweighted results

WITNESS Lepto	MAT positive (*n*)	MAT negative (*n*)
Positive	TP: 49 (positive group)	FP: 10 (borderline group)
Negative	FN: 1 (positive group)	TN: 25 (borderline group),
		50 (negative group)

**Table:** (b) Weighted data used to calculate Se and Sp

WITNESS Lepto	MAT positive (*n*)	MAT negative (*n*)
Positive	TP: 33.075	FP: 6.55
Negative	FN: 0.675	TN: 94.45
	Sensitivity: 98%	Specificity: 93.5%

MAT: microscopic agglutination test; Se: sensitivity; Sp: specificity; TP: true positive; TN: true negative; FP: false positive; FN: false negative.
